# Scandium doping brings speed improvement in Sb_2_Te alloy for phase change random access memory application

**DOI:** 10.1038/s41598-018-25215-z

**Published:** 2018-05-01

**Authors:** Xin Chen, Yonghui Zheng, Min Zhu, Kun Ren, Yong Wang, Tao Li, Guangyu Liu, Tianqi Guo, Lei Wu, Xianqiang Liu, Yan Cheng, Zhitang Song

**Affiliations:** 1 State Key Laboratory of Functional Materials for Informatics, Shanghai Institute of Micro-system and Information Technology, Chinese Academy of Sciences, Shanghai, 200050 China; 2 School of Physical Science and Technology, ShanghaiTech University, Shanghai, 201210 China; 3 Institute of Microstructure and Property of Advanced Materials, Beijing University of Technology, Beijing, 100124 China; 4 Present Address: Key Laboratory of Polar Materials and Devices, Ministry of Education, East China Normal University, Shanghai, 200062 China

## Abstract

Phase change random access memory (PCRAM) has gained much attention as a candidate for nonvolatile memory application. To develop PCRAM materials with better properties, especially to draw closer to dynamic random access memory (DRAM), the key challenge is to research new high-speed phase change materials. Here, Scandium (Sc) has been found it is helpful to get high-speed and good stability after doping in Sb_2_Te alloy. Sc_0.1_Sb_2_Te based PCRAM cell can achieve reversible switching by applying even 6 ns voltage pulse experimentally. And, Sc doping not only promotes amorphous stability but also improves the endurance ability comparing with pure Sb_2_Te alloy. Moreover, according to DFT calculations, strong Sc-Te bonds lead to the rigidity of Sc centered octahedrons, which may act as crystallization precursors in recrystallization process to boost the set speed.

## Introduction

Recently, with the increasing requirements of dealing with tons of information on the Internet, intelligent devices and portable computers are developing very fast. Among the numerous nonvolatile memories that are used in commercial devices, Flash memory is the mainstream technology. Nevertheless, with the feature size of integrated circuit scaling down, it is approaching its dimension extreme^1^. Hence, it is necessary to develop new nonvolatile memory technology. As one of promising candidate technologies, phase change random access memory (PCRAM) possesses superior properties, such as low cost, high scalability and high integration level^2,3^. It has been published that PCRAM can still realize reversible switching performance in a tiny cell (<2 nm)^4^. Thus, PCRAM is a very prospective storage technology to reform the current nonvolatile memory market.

PCRAM works through distinguishing the resistance difference between amorphous (high resistive) and crystalline (low resistive) states^5^. Phase change material is the key of PCRAM. Hence, the phase transition process with different mechanisms^6–8^ has been a hot research area for several years. For each of these materials, Ge_2_Sb_2_Te_5_ (GST) alloy is the most mature material with good comprehensive properties. Both vacancies and Te have been considered to play an important role in the fast phase transition process of GST^9^. As commercialized phase change material, GST still has some flaws like relatively slow crystallization speed (~50 ns)^10^ and low data retention temperature (~85 °C)^11^, and so on, which hinder its widely applications in PCRAM. Hence, researchers never stop developing new materials for getting better and better properties. Sb_2_Te alloy as a basic member of phase change material family, shows high crystallization speed, which is attributed to its growth dominated crystallization mechanism^12^. However, poor amorphous stability and data retention cut off the possibility of its potential application in PCRAM without optimization^13^. In some previous studies, doping is beneficial to increase the crystallization temperature of base materials, such as doping N^14,15^, Cu^16^ and W^17^. But after doping, phase separation is always accompanied, impeding the further improvement of crystallization speed, deteriorating device performance, and decreasing cycling times^18^. Ti doping was also found to increase the crystallization speed owing to Ti centered atomic motifs in both amorphous and crystalline state^19^. Later calculation work predicts that Y dopants match well with parent Sb_2_Te_3_ structure and increase the stability of Sb_2_Te_3_ in the phase change process^20^. Furthermore, a recent report from Science points out that Sc-doped Sb_2_Te_3_ phase change material without phase separation has a very rapid SET speed reaching up to 700 picoseconds^21^ Among the equilibrium phases of Sb-Te system, Sb_2_Te alloy has >50 °C higher crystallization temperature than Sb_2_Te_3_ one. Hence, in this paper, Sc element was also been chosen as a dopant into Sb_2_Te alloy. We hope that through the adjustment of substrate material, the new one could have better thermal stability, and also useful for speed improvement in Sb_2_Te alloy.

## Results

**Figure 1:**
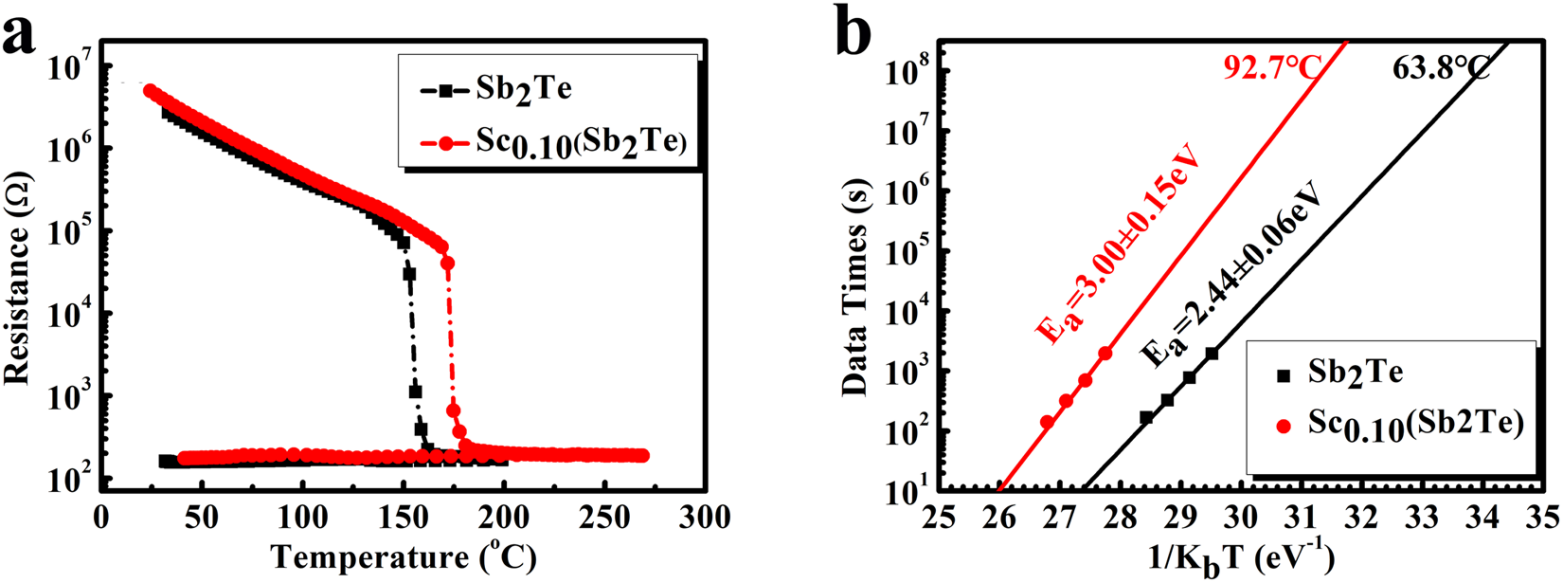
(**a**) R-T curves for Sb_2_Te and Sc_0.1_Sb_2_Te films with a heating rate of 20 °C/min. (**b**) The Arrhenius extrapolation plots of 10-year data retention time versus 1/K_b_T for Sb_2_Te and Sc_0.1_Sb_2_Te films.

The sheet resistance as a function of temperature (R-T) for Sb_2_Te and Sc_0.1_Sb_2_Te films (~100 nm) was measured to clarify the influence of doping on the thermal stability as depicted in Fig. 1(a). The sharp drop of the resistance happens around the crystallization temperature (*T*_*c*_), which significantly shifts to higher temperature after doping Sc. According to the derivative of logarithmic sheet resistance with respect to temperature (dlgR/dT), the *T*_*c*_ of Sb_2_Te and Sc_0.1_Sb_2_Te films are estimated to be 156.1 °C and 174.9 °C, respectively, indicating better amorphous stability after Sc doping. Figure 1(b) shows the 10-year data retention characteristics for Sb_2_Te and Sc_0_._1_Sb_2_Te films. Based on the Arrhenius equation: *t* = *τ*·exp(*E*_*a*_/*k*_*B*_*T*), where *t* is the 50% criterion failure time, τ is the proportional time constant, ${E}_{a}$ is the crystallization activation energy, ${k}_{B}$ is the Boltzmann constant, *T* is the absolute temperature. After Sc doping, the activation energy ${E}_{a}$ increases from 2.44 eV to 3.00 eV. By extrapolating the data retention time to 10 years, the data retention temperature for Sc_0.1_Sb_2_Te film is estimated to be 92.7 °C, demonstrating a better thermal stability than that of Sb_2_Te (63.8 °C) and conventional GST (85 °C) alloy. Apart from better thermal stability, four orders of magnitude resistance difference leave enough margins for identifying the high resistance and low resistance states.

**Figure 2:**
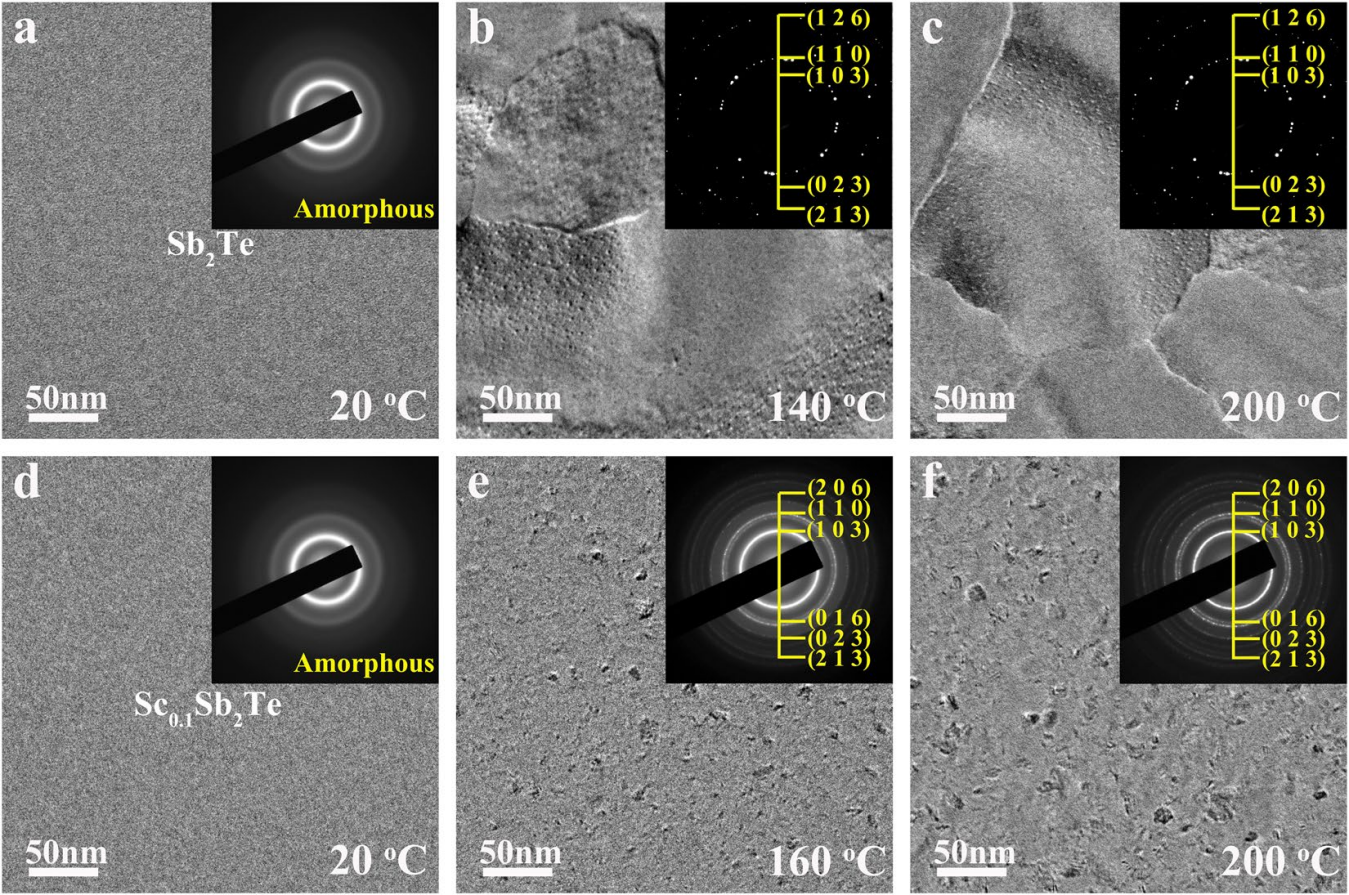
TEM BF images and their corresponding SAED patterns reveal the *in-situ* thermally-induced crystallization processes of (**a**–**c**) Sb_2_Te and (**d**–**e**) Sc_0.1_Sb_2_Te films at different temperature.

From microstructural side, thermally-induced phase transition processes were investigated by *in-situ* transmission electron microscope (TEM) technique for Sb_2_Te and Sc_0.1_Sb_2_Te films (~15 nm) with a heating rate of 10 °C/min. Figure 2 shows TEM bright-field (BF) images and the corresponding selected area electron diffraction (SAED) patterns for Sb_2_Te (a-c) and Sc_0.1_Sb_2_Te (d-f) films at different temperature. Pure Sb_2_Te film starts to crystallize with an explosive crystal growth at 140 °C (Fig. 2b), and the grain size is in about several hundred nanometers scale. After the temperature increases to 200 °C (Fig. 2c), the grain size of Sb_2_Te film is almost the same comparing with Fig. 2b, because its crystallization process has already finished around 140 °C. As for Sc_0.1_Sb_2_Te film, it starts to crystallize at 160 °C with numerous nanocrystals (<~10 nm) as shown in Fig. 2d. Though these nanocrystals grow a little (<~15 nm) as temperature rises up to 200 °C (Fig. 2e), the grain size of Sc_0.1_Sb_2_Te is still much smaller than that of Sb_2_Te after crystallization. In addition, both of the SAED patterns in Fig. 2c,f can be indexed as hexagonal (h-) Sb_2_Te structure (JCPDS No. 80–1722). No extra diffraction rings appear in Fig. 2f, which demonstrates that Sc_0.1_Sb_2_Te film is a single h-phase without phase separation. The XRD result of crystallized Sc_0.1_Sb_2_Te film, as shown in Fig. S1, further confirms that Sc_0.1_Sb_2_Te has the same structure as Sb_2_Te. That is, Sc doping significantly affects the crystallization behavior of Sb_2_Te film without forming any new phase or new structure. Beyond that, a crystallized film with three times more of Sc doping level, as much as 11% (Sc_0.4_Sb_2_Te), was investigated to inspect the distribution of Sc atoms by using of STEM-EDS mapping in TEM as shown in Fig. S2. Even at a higher doping level, the crystalline structures of Sc_0.4_Sb_2_Te and Sc_0.1_Sb_2_Te remain the same from SAED pattern side. This EDS results give more direction on uniform distributed Sc, Sb and Te elements without obvious phase separation appearing in nanometer scale.

**Figure 3:**
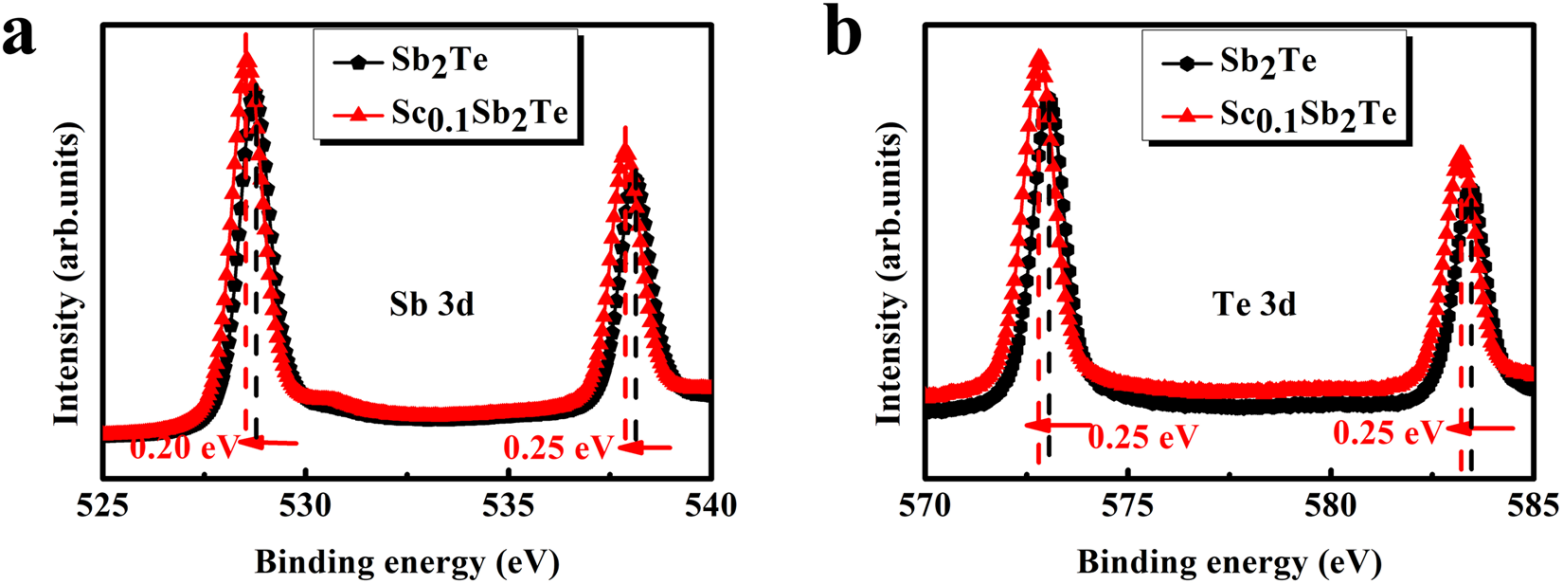
XPS spectra for annealed Sb_2_Te and Sc_0.1_Sb_2_Te films: (**a**) Sb 3d, and (**b**) Te 3d.

In order to understand the interplay between Sc atoms and Sb_2_Te lattice, XPS experiment was applied to investigate the bonding state of crystallized Sb_2_Te and Sc_0.1_Sb_2_Te films. Figure 3 shows the binding energy of Sb 3d and Te 3d core levels for Sb_2_Te and Sc_0.1_Sb_2_Te films. The C *1 s* peak at 284.8 eV is used as a reference. After Sc doping, both peaks of Sb 3d shift to lower energies (~0.2 eV for Sb 3d_5/2_, ~0.25 eV for Sb 3d_3/2_). Similar results are observed in the binding energies for Te 3d (~0.25 eV for both Te 3d_5/2_ and Te 3d_3/2_). Usually, binding energy will decrease when an atom bonds to another one with a lower electro-negativity. Since the electro-negativity of Sc (1.36) is smaller than that of Sb (2.05) and Te (2.12), Sc atoms is very likely to bond with Sb and Te atoms in Sc_0.1_Sb_2_Te film after crystallization, resulting in the decrease of binding energy for Sb and Te elements. Considering that the electro-negativity difference (ΔS) of Sc-Te and Sc-Sb is much bigger than Sb-Te, and a large ΔS between two atoms would increase nucleation probability^22,23^. More nuclei are likely to generate after Sc doping, and the intergrowth of nuclei produces more grain boundaries, which will suppress the subsequent crystal growth significantly. This may be contributing to explain the much smaller grain size distribution after Sc doping as shown in Fig. 2f.

**Figure 4:**
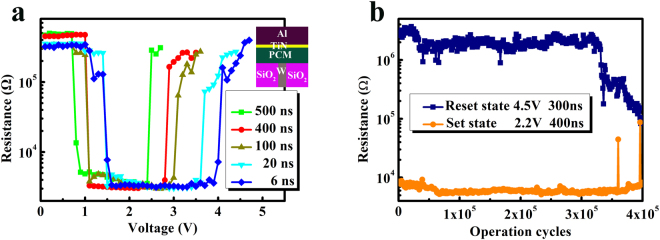
(**a**) Resistance-Voltage characteristics of Sc_0.1_Sb_2_Te based PCRAM device with difference voltage pulse widths. The inset depicts the schematic diagram of the T-shaped PCRAM cell structure. (**b**) Reversible switching characteristic of the device.

Anyway, good device performances are the key to application. Figure 4a shows the resistance-voltage curves of Sc_0.1_Sb_2_Te alloy based PCRAM cell with different pulse widths (the falling edge of the voltage pulse is 3 ns). Both set and reset voltages slightly shift to a higher value when the pulse width decreases. But most of all, even 6 ns electrical voltage pulse can still induce reversible phase transformation in this PCRAM device. Comparing to conventional GST (crystallization speed of ~ 50 ns)^10^ and Sb_2_Te (crystallization speed of ~ 20 ns)^13^, Sc_0.1_Sb_2_Te based PCRAM cell exhibits faster operation speed. Besides, endurance up to 3.3 × 10^5^ cycles without failure (Fig. 4b) also demonstrates that Sc_0.1_Sb_2_Te alloy has great potential for PCRAM application^24^.

## Discussion

To further verify the location of doped Sc atoms, Ab initial method was carried out to theoretically predict the most probable site by calculating the formation energy (${E}_{f}$) in each site. Rather than simulating the exact experiment composition, we introduce a single Sc atom at various lattice sites in a Sb_2_Te supercell with lattice parameter of 12.816◊12.816◊ 17.633(Å^3^) to evaluate the effects of doped Sc atom. In the Sb_2_Te supercell, there are seven possible dopant sites for Sc atoms, Sb_1_, Sb_2_, Sb_3_, Te_1_, Te_2_ and In_1_, In_2_ (shown in Fig. S3a). Sb and Te with subscript mean the substitution doping in which the Sc atom replaces Sb or Te, whereas In1 and In2 mean the Sc atom enters the interstitial site. The formation energy of each structure after relaxed was calculated and shown in Fig. S3b. The ${E}_{f}$ was obtained according to the following equation:

$${E}_{f}=({E}_{Sc \mbox{-} doped}+{E}_{Sb/Te})-({E}_{un \mbox{-} doped}+{E}_{Sc})$$

**Figure 5:**
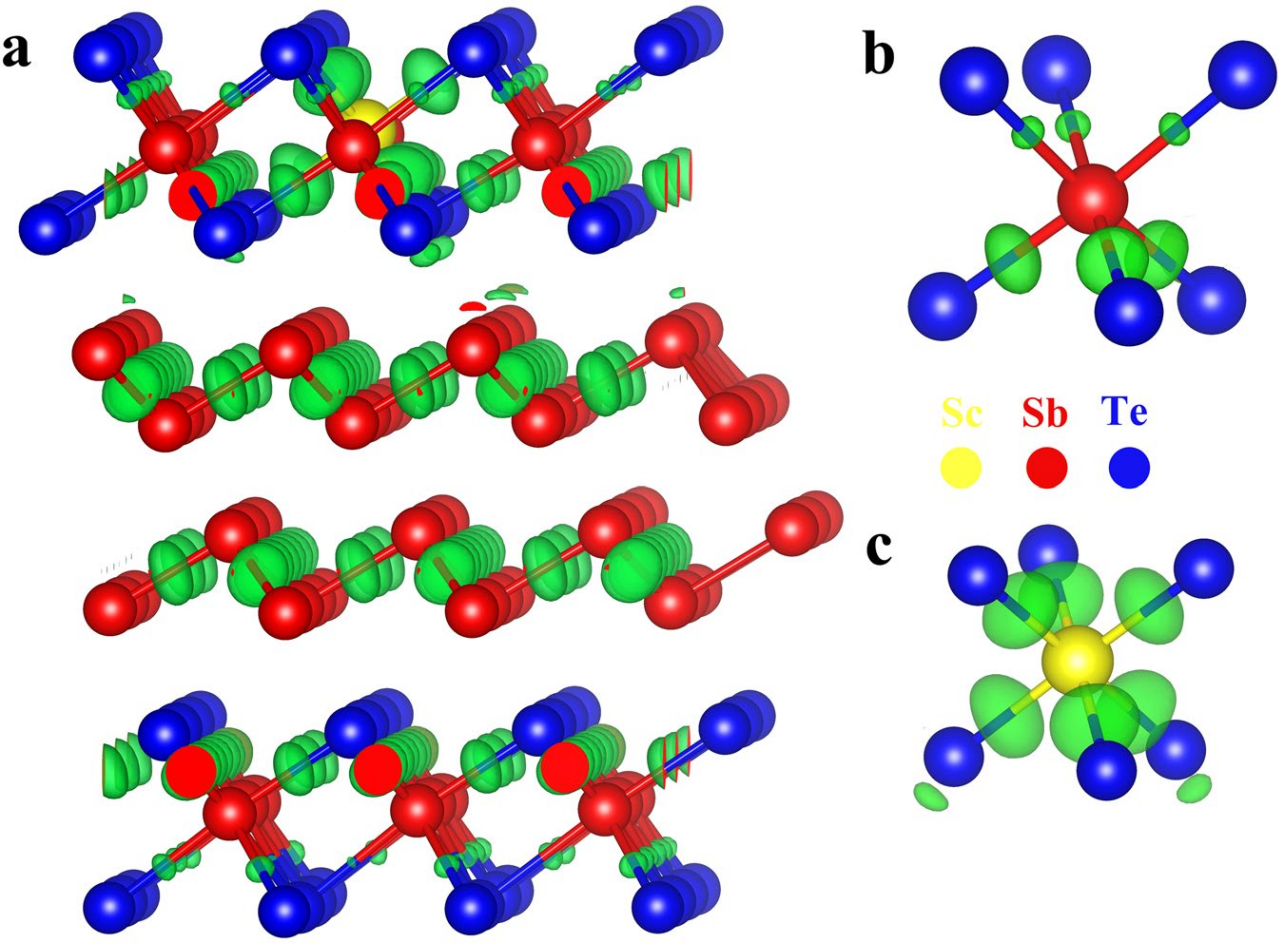
Bonding chemistry of Sc doped Sb_2_Te when Sc atom substitutes site 1. (**a**) charge density difference in the quintuple layers of Sc doped Sb_2_Te, the differences are calculated with respect to the charge density of isolated atoms, the isosurface (transparent green area) is fixed at +0.0046 a_0_^−3^ (a_0_ = bohr). (**b**,**c**) are the Sb and Sc centered octahedron, respectively.

Here ${E}_{un \mbox{-} doped}$ and ${E}_{Sc \mbox{-} doped}$ denote the total energies of the relaxed structure before and after Sc doping. ${E}_{{Sc}}$ denotes the chemical potential of doped Sc, ${E}_{{Sb}/{Te}}$ denotes the chemical potential of Sb or Te being replaced, while it is zero for interstitial doping. As shown in Fig. S3b, the ${E}_{f}$ for Sb_1_ is −2.482/2.583 eV for Sb/Te rich, which is much lower than all of the other conditions. Thus, Sb_1_ is the most energetically favorable position for the doped Sc atom. To identify the bonding information of Sc_0.1_Sb_2_Te when Sc substitutes Sb_1_, the charge density difference (CDD) of relaxed structure was illustrated in Fig. 5. Their corresponding 2D charge density plot was shown in Fig. S4, which shows that Sc and Sb are bound with Te through a bond point^25^. In order to show the chemical environment of Sc, we present the nine layers that exist in the Sb_2_Te h-structure along C axis. As shown in Fig. 5b,c, there is only tenuous charge present in three of the Sb-Te bonds which is in distinct contrast with the noticeable charge accumulation at the bond center between Sc and Te. The original Sb-centered octahedron shows three strong (3.045 Å) bonds and three weak bonds (3.159 Å). However, the Sc-centered octahedron shows six identical strong bonds (2.98 Å), the strong Sc-Te bonds lead to the rigidity of Sc-centered octahedrons. Even they may not necessarily be intact in the melt-quenched amorphous phase, yet the Sc-centered octahedrons can still be the subcritical embryos owe to their lowest formation energy. So the reconstruction of Sc-centered octahedrons is more advantageous than that of Sb-centered motifs in the recrystallization process. The existence of large amounts of precursors will refine the crystalline size, thus increase the grain boundaries which will accommodate more stress produced in phase change process of PCRAM device. Moreover, smaller grain size will increase the interface-area-to-volume ratios to facilitate the hetero-crystallization at the grain boundaries, further accelerating the crystallization speed. This may explain why the SET speed of Sc_0.1_Sb_2_Te based PCRAM device (6 ns) is faster than Sb_2_Te based one (20 ns). However, after 3.3 × 10^5^ cycles, the non-uniform electronic fields in the active mushroom shape area might lead to a reallocation of elements, thus appear Sb_2_Te large grains that can result in device failure.

Comparing with Sc_0.2_Sb_2_Te_3_ material, Sb_2_Te alloy in this study was choosing as the parent material instead of Sb_2_Te_3_ alloy, considering the Sb_2_Te’s thermal stability is better. Sc_0.2_Sb_2_Te_3_ material can change from amorphous state to face centered cubic (f-) phase, and then to the stable h-phase with the increase of temperature. The 700 picoseconds set speed^21^ only involves phase change process between amorphous and f-phase, which is a metastable state like f-GST phase. Avoiding f-to-h phase transition is very important in PCRAM device, with an emphasis on preventing grain growth^26^. Hence, in this paper, Sc_0.1_Sb_2_Te material can reach 6 nanoseconds set speed without intermediate phase, benefiting from its strong Sc-centered cluster. It has high-speed and good thermal stability together, which may point out some direction for the future design of PCRAM devices.

## Conclusion

In this paper, Sc doped Sb_2_Te film was investigated to verify its application in PCRAM. After Sc doping, the thermal stability of Sb_2_Te alloy was improved, and the 10-year data retention time was increased. The crystalline Sc_0.1_Sb_2_Te film exhibits a single phase without phase separation. Sc_0.1_Sb_2_Te based PCRAM cell can still realize stable reversible switching behaviors even at 6 ns. Two orders of resistance difference between set and reset state makes it easy to distinguish “0” and “1”. Furthermore, endurance up to 3.3 × 10^5^ cycles makes Sc_0.1_Sb_2_Te a promising material for PCRAM application.

## Methods

Sb_2_Te and Sc doped Sb_2_Te films was deposited on SiO_2_/Si (100) substrates and carbon coated TEM grid by co-sputtering Sc and Sb_2_Te targets using RF sputtering system at room temperature. The composition of the deposited films, BF images and SAED patterns were characterized by JEOL-2100F TEM with energy dispersive spectroscopy (EDS). The bonding situation of Sb_2_Te and Sc_0.1_Sb_2_Te alloy was evaluated by X-ray photoelectron spectroscopy (XPS) with Al Kα radiation. T-shaped PCRAM cells with a tungsten bottom electrode (190 nm in diameter) were fabricated using 130 nm CMOS technology. Afterwards, Sc_0.1_Sb_2_Te film (about 55 nm), TiN film (10 nm) and Al top electrode (300 nm) were sequentially deposited. Resistance-voltage curves and programming cycles were monitored with Keithley 2400 and Tektronix AWG 5002B. Calculations in this work was investigate by using density functional theory (DFT)^27^. The Vienna Ab-initio Simulations Package (VASP)^28^ was used for calculations. The projector augmented wave (PAW)^29^ pseudopotentials were used to describe electron-ion interactions. For the exchange-correlation energies between electrons, the Perdew-Burke-Ernzerhof (PBE)^30^ function was employed. The energy cut offs were chosen to be 450 eV and 350 eV for relaxation and static calculation. A supercell containing 3◊3◊1 unit cells of Sb_2_Te was constructed for relaxation. The 5◊5◊3 K point mesh with Gamma centered was used. The relaxation was performed until the total energy converged to within 1 meV.

## Electronic supplementary material

Supplementary Information
