# Supplementary material for: Scandium doping brings speed improvement in Sb2Te alloy for phase change random access memory application
Source: Sci Rep. 2018 May 1;8:6839. doi: 10.1038/s41598-018-25215-z (PMC5931567; doi:10.1038/s41598-018-25215-z)
Supplement: Supplementary file 1 — Supplementary Information [file 41598_2018_25215_MOESM1_ESM.docx]

**Scandium doping brings speed improvement in Sb_2_Te alloy for phase change random access memory application**

Xin Chen^1,2^, Yonghui Zheng^1^, Min Zhu^1^, Kun Ren^1^, Yong Wang^1^, Tao Li^1^, Guangyu Liu^1^, Tianqi Guo^1^, Lei Wu^1^, Xianqiang Liu^3^, Yan Cheng^1^^*^**^†^**, Zhitang Song^1,2^

*^1^State Key Laboratory of Functional Materials for Informatics, Shanghai Institute of Microsystem and Information Technology, Chinese Academy of Sciences, Shanghai 200050, China*

*^2^School of Physical Science and Technology, Shanghai Tech University, Shanghai* *201210, China*

*^3^Institute of Microstructure and Property of Advanced Materials, Beijing University of Technology, Beijing 100022, China*

^*^*Corresponding author. E-mail:* *ycheng@ee.ecnu.edu.cn.*

*^†^Present address: Key Laboratory of Polar Materials and Devices, Ministry of Education, East China Normal University, Shanghai 200062, China*

**Supplementary 1**

**
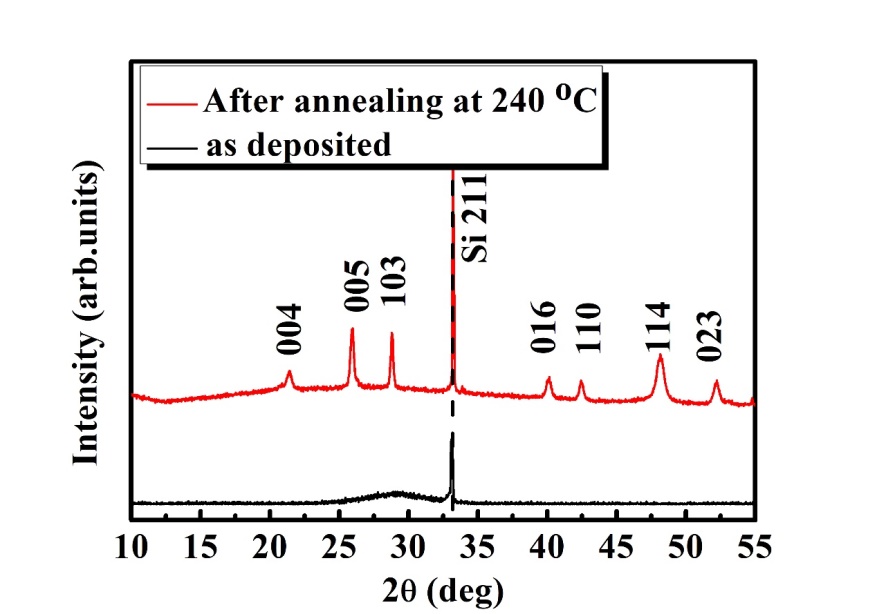
**

Figure S1. The XRD results of Sc_0.1_Sb_2_Te films before and after annealing for five minutes at 240 ^o^C. The as deposited film is amorphous state. Crystallized Sc_0.1_Sb_2_Te film shows hexagonal structure, which is consistent with Sb_2_Te (JCPDS No. 80-1722).

**Supplementary 2**

**
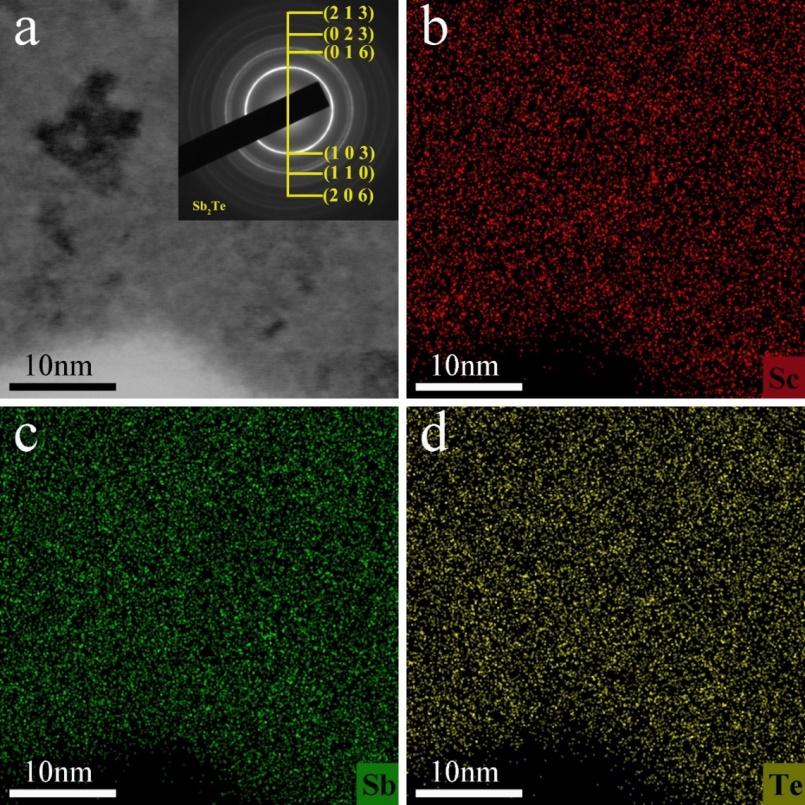
**

Figure S2. The bright-filed STEM image and corresponding EDS distribution for Sc, Sb and Te elements after crystallization. Fig. S2 shows the EDS mapping of Sc doped Sb_2_Te crystallized film after heating to 260 ^o^C. The white background is the empty area of the film as shown in the lower area of Fig. S1a. The uniform distribution of Sc, Sb and Te elements is observed from Fig. S2 b-d. In order to achieve an efficient signal counts, a crystallized film with Sc doping level as much as 11% is used instead of 3% since its crystal structure is the same as Sc_0.1_Sb_2_Te.

**Supplementary 3**


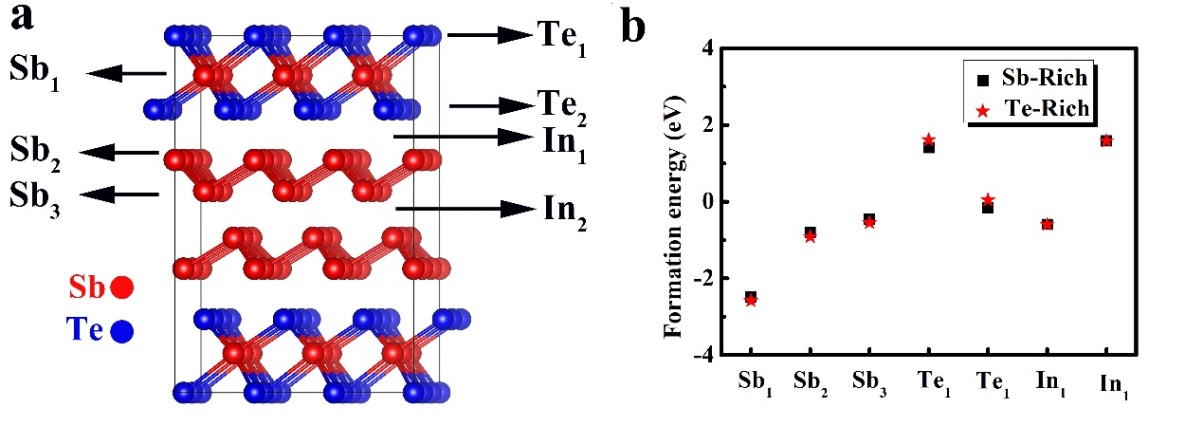


**Figure S3.** (a) The supercell model of Sb_2_Te with a layered structure: Te_1_-Sb_1_-Te_2_-Sb_2_-Sb_3_-Sb_3_-Sb_2_-Te_2_-Sb_1_, depicting three inequivalent Sb sites, Sb_1_, Sb_2_ and Sb_3_, two inequivalent Te sites, Te_1_ and Te_2_, and two possible interstitial sites, In_1_ and In_2_. (b) their corresponding (shown in parentheses) defect formation energy when Sc atom substitutes Sb_1_, Sb_2_, Sb_3_, Te_1_, Te_2_ or enters the interstitial site, In_1_ and In_2_, respectively.

**Supplementary 4**

**
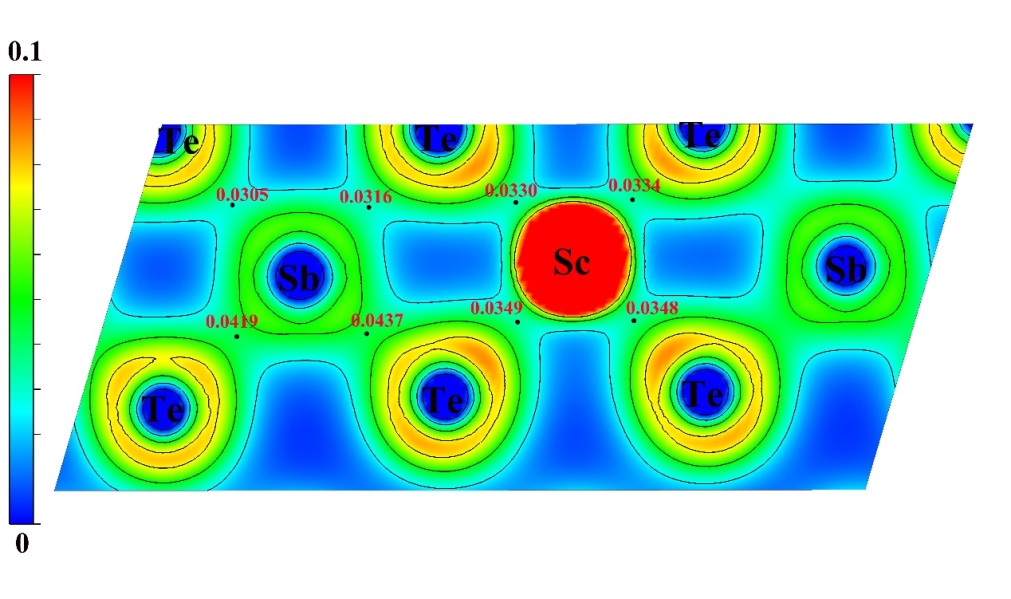
**

Figure S4. The charge density plot of the same atomic structure shown in Figure 5, the atoms near Sc was chosen for clarification. The image shows that Sc and Sb are bound with Te through a bond point (saddle point).
